# New records and species of deep-sea squat lobsters (Galatheoidea, Munidopsidae) from the Hawaiian Archipelago: an integrative approach using micro-CT and barcodes

**DOI:** 10.7717/peerj.14956

**Published:** 2023-03-08

**Authors:** Paula C. Rodríguez Flores, Kareen E. Schnabel

**Affiliations:** 1 Department of Organismic and Evolutionary Biology, Museum of Comparative Zoology, Harvard University, Cambridge, Massachusetts, United States; 2 Marine Biodiversity & Biosecurity, National Institute of Water & Atmospheric Research, Wellington, New Zealand

**Keywords:** 3D imaging, Crustaceans, Anomura, Morphology, Molecular taxonomy, Phylogenetics, Systematics

## Abstract

The Hawaiian Archipelago remains extensively under-sampled for many marine invertebrate taxa, including squat lobsters. During the last few years, several deep-sea expeditions carried out in the Pacific Ocean have conducted opportunistic collections of specimens and image data from the vicinity of Hawai’i. Here we describe a new species: *Munidopsis hawaii* **sp. nov**. and provide new records for Munidopsidae in the Archipelago and its associations. We illustrate and describe the new species using an integrative approach including micro-CT 3D imaging. Phylogenetic analyses of the species collected from seamounts from Hawai’i indicate that the new species represents a divergent lineage compared to morphologically similar species such as *M. dispar* and *M. papanui*. We also study the genetic distances for the species recorded in Hawai’i and other populations of the same species in the adjacent West Pacific. Three species are now known in the Hawaiian region. We also compiled identifications from images captured with ROVs in the area. These observations suggest that munidopsid species are common in the deep sea of Hawaiian waters below 1,000 m.

## Introduction

The Hawaiian Archipelago includes a high diversity of deep-sea habitats including ridges, banks, seamounts, submarine volcanos, and an active mid-plate hydrothermal system (Karl et al., 1988; Baco, 2007). It is considered a hotspot for deep-sea coral diversity (Rogers et al., 2007), but it remains largely under-sampled for many other invertebrate taxa (Baco, 2007).

Until now, 15 species of squat lobsters (superfamilies Galatheoidea and Chirostyloidea) have been formally recorded or described from the ocean around the Hawaiian Islands. Eight species belong to the superfamily Chirostyloidea Ortmann, 1892, including three species of *Uroptychus* Henderson, 1888, three species of *Eumunida* Smith, 1883, *Pseudomunida fragilis* Haig, 1979 and *Sternostylus hawaiiensis* (Baba, 1977) species. Galatheoidea, includes seven species from Hawaii: *Galathea spinosorostris* Dana, 1852, *Phylladiorhynchus integrirostris* (Dana, 1852), *Garymunida normani* (Henderson, 1885), *Babamunida debrae* Baba, 2011, *Babamunida kanaloa* Schnabel, Martin & Moffitt, 2009, *Paramunida hawaiiensis* (Baba, 1981) and *Trapezionida heteracantha* (Ortmann, 1892; Baba, 1981; Baba et al., 2008; Castro, 2011; Schnabel & Ahyong, 2019); although the records corresponding to some of these species are dubious (Schnabel, Martin & Moffitt, 2009). Except for *Pseudomunida fragilis* which was found at a depth of *ca*. 1,000 m (Haig, 1979), all these records correspond to shallow and shelf species. Very little is so far known about deep-sea squat lobsters of bathyal and abyssal depths, with the family Munidopsidae currently absent in the Hawaiian Archipelago (Baba et al., 2008; Schnabel, Martin & Moffitt, 2009; Macpherson et al., 2010). Munidopsid species constitute the deepest dwellers among squat lobsters, with the most diverse genus, *Munidopsis* Whiteaves, 1874, almost absent from subtidal and shelf depths (Baba, 2005; Schnabel et al., 2011). Some of the species in the genus are distributed worldwide at abyssal depths up to *ca*. 5,200 m (Macpherson, 2007; Jones & Macpherson, 2007; Schnabel et al., 2011).

During the exploration of the Papahānaumokuākea Marine National Monument (PMNM) carried out by the RV NOAA Okeanos Explorer, several deep-sea squat lobsters were observed in the vicinity of the Hawaiian Islands, including those in genera *Munida *sensu* lato*, *Munidopsis*, *Uroptychus*, *Gastroptychus* Caullery, 1896 and *Pseudomunida fragilis* (*e.g*., Kennedy & Mcletchie, 2016). Photographs were obtained during this expedition, and Wicksten (2020) reported on several *in situ* observations of chirostyloid squat lobsters and their faunal associations from north of the Hawaiian Archipelago. Several photographs of munidopsid species from ROV dives around the Hawaiian Archipelago and Johnston Atoll were made available online through the NOAA Ocean Exploration data archive (https://oceanexplorer.noaa.gov/data/access/access.html) and in the NOAA Ocean Exploration Benthic Deepwater Animal Identification Guide (NOAA Ocean Exploration Benthic Deepwater Animal Identification Guide, http://oceanexplorer.noaa.gov/okeanos/animal_guide/animal_guide.html). In 2018, an expedition of the EV Nautilus mapped several seamounts of poorly explored areas of the PMNM and opportunistically collected some squat lobsters using remotely operated vehicles (ROVs). The material was later deposited in the Invertebrate Zoology collection in the Museum of Comparative Zoology (MCZ) at Harvard University, Cambridge, MA. Here, we revise the available specimens using morphological and molecular characters and report a new species, which is described and illustrated using micro-CT 3D imaging. We also reconstructed the systematic position of the new species using two mitochondrial and one nuclear gene. These provide the first report of the family Munidopsidae for the Hawaiian Archipelago, which includes five *Munidopsis* species and *Galacantha rostrata* (Milne-Edwards, 1880). The presence of additional species was recorded by ROVs images from the sources listed above, but physical specimens for a detailed morphological examination would be needed to confirm the identity.

## Materials and Methods

### Morphological examination

Specimens were examined using a Leica MZ 12.5 stereomicroscope. Drawings were made using a camera lucida and these drawings were digitalized using a Wacom Intuos Pro tablet. The terminology used for the species descriptions follows Baba et al. (2009) and Baba, Ahyong & Macpherson (2011). Size is indicated by the postorbital carapace length (PCL). Rostrum length is measured from the base (frontal margin) to the distal tip of the rostrum; rostrum width is taken as the rostrum’s base width. Measurements of appendages are taken on dorsal (pereopod 1) and lateral (pereopods 2–4) midlines. Ranges of morphological and meristic variations are included in the description. Abbreviations used are the following: Mxp = maxilliped; P1 = pereopod 1 (cheliped); P2–4 = pereopods 2–4 (walking legs 1–3); M = male; F = female; ov. = ovigerous. The examined specimens are deposited in the Invertebrate Zoology collection in the Museum of Comparative Zoology (MCZ), Cambridge, MA.

### Micro-CT imaging

The holotype of the new species was mounted in a plastic vial and dried. A falcon tube and polypropylene cotton or synthetic cotton were used to avoid the specimen moving, and the container was sealed with parafilm.

The Micro-Computed Tomography (micro-CT) scan was performed using a SkyScan 1273 scanner (Bruker MicroCT, Kontich, Belgium). The scanner is equipped with a Hamamatsu 130/300 tungsten X-ray source 40–130 kV and a flat-panel X-ray detector with 6-megapixel (3,072 × 1,944). We chose the following scanning parameters: source current = 100 µA, source voltage = 75 kV, exposure time = 1,000 ms, frames averaged = 2–6, rotation step = 0.3, frames acquired over 180° = 960, filter = no, binning = no, flat field correction = activated. The scanning time was around 120 min. We reconstructed the scan using the software NRecon 1.6.6.0, Bruker MicroCT, Kontich, Belgium. To enhance image contrast and compensate for the ring and streak artifacts the following reconstruction parameters were set: smoothing = no, ring artifact correction = 5–11, and beam hardening correction = activated. 3D rendering images and segmentation were performed by using Amira software (Thermo Fisher Scientific, Waltham, MA, USA).

### DNA extraction, amplification, and sequencing

Specimens were dissected under a stereomicroscope. Tissue subsampled from the 5^th^ pereiopod was digested overnight. For DNA extraction we used the DNeasy Blood & Tissue kit (Qiagen) following the manufacturer’s protocol. Partial sequences of the mitochondrial gene cytochrome *c* oxidase subunit I (COI), the 16S ribosomal RNA, and the nuclear 28S ribosomal RNA were amplified by polymerase chain reaction (PCR) using the combination of the following primers: LCO 1490/HCO 2198 (Folmer et al., 1994), often combining universal primers with internal specific primers for squat lobsters, *i.e*., tenuiCOIFwint (Rodríguez-Flores et al., 2019); 16SAR/16SBR (Palumbi, 1991), or 16S1471/16S1472 (Crandall & Fitzpatrick, 1996); and 28SBR/28Sphyf2 (Palero, 2008, Rodríguez-Flores et al., 2022a). For the DNA amplification, PuReTaq Ready-To-Go (RTG) PCR Beads (Cytiva) were employed, following the following cycle conditions: 5 min of initial denaturation at 95 °C followed by 35 cycles of 30 s denaturation at 95 °C, 45 s of annealing at 45–50 °C, 1 min of extension at 72 °C, and a 10 min final extension at 72 °C. Purification of the amplicons was carried out using ExoSAP-IT (Affymetrix). Sanger sequencing of both forward and reverse strands was performed by GeneWiz (Cambridge, UK). Forward and reverse DNA sequences obtained for each specimen were checked and assembled using Sequencher v.5.4 (Gene Codes Corporation, Ann Arbor, MI, USA), which was also used to check for the presence of pseudogenes. The online web tool EXPASY (https://web.expasy.org/translate/) was also used to translate the nucleotide sequences to amino acids under the invertebrate mitochondrial genetic code. Ribosomal gene sequences were aligned with MAFFT (Katoh et al., 2002), using the iterative method L-INS, recommended for sequences with conserved domains flanked by long variable regions, like in the dataset here analyzed, especially for the 28S rRNA gene. *A posteriori* manual checking and correction of the alignments were carried out in AliView (Larsson, 2014). The alignment of the concatenated gene fragments was built in PAUP v.4.0a (build 169) (Swofford, 2002), which was also used to calculate the uncorrected genetic distance (*p*) between the new species and its closest relatives.

### Phylogenetic analyses

Phylogenetic analyses were performed using the concatenated dataset of the three genes. We first retrieved sequences from previous publications from NCBI which included morphologically related species to the species here examined (Rodríguez-Flores, Macpherson & Machordom, 2018; Dong, Gan & Li, 2021; Rodríguez-Flores, Macpherson & Machordom, 2022b). Taxa information and GenBank accession numbers are summarized in Table 1. *Leiogalathea ascanius* Rodríguez-Flores, Macpherson & Machordom, 2019 was selected as the outgroup for Munidopsinae (*sensu* Ahyong, Andreakis & Taylor, 2011). We ran BEAST v2.6.3 (Bouckaert et al., 2014) for the Bayesian inference (BI) analyses, and Maximum likelihood analyses (ML) to compare both ML and BI results. A partition scheme by gene was selected, according to the results of Model Selection in W-IQ-TREE Online v.1 (Trifinopoulos et al., 2016) based on a Bayesian information criterion (BIC). For the BI analyses, we set the parameters in BEAUti v2.6.3 (Bouckaert et al., 2014) choosing a relaxed clock lognormal for the molecular clock with clock.rate fixed = 1 since we did not intend to estimate the time of divergence of the genes. The tree prior selected was a Yule Model. Four Markov Chains Monte Carlo (MCMC) were run for 1 × 10^7^ generations and sampling trees and parameters every 1,000 generations for the estimation of the posterior probabilities. The initial 25% of the generations were discarded as burn-in. The convergence of the chains was checked using Tracer v1.7.1 (Rambaut et al., 2018). The ML tree was inferred with W-IQ-TREE Online v.1 (Trifinopoulos et al., 2016). Bootstrap support values were calculated with 1000 pseudoreplicates and the other parameters were set as default. Nodes were considered supported when bootstrap values (Bs) were higher than 70% and posterior probability (pP) higher than 0.95. The phylogenetic trees were plotted and edited in FigTree v1.4.4. Posterior probabilities from the BI and bootstrap support from ML were included in the final tree.

**Table 1:** Specimens analyzed. List of taxa used for this study, including GenBank accession numbers, and reference associated.

Code	Species ID/GenBank accession numbers	COI	16S	28S	Reference
MNHN-IU-2014-13772	*Leiogalathea ascanius*	MK140849	MK140885	MN839902	Rodríguez-Flores, Macpherson & Machordom (2019)
Mp9	*Munidopsis polymorpha*	ON886904	OP942424	ON858165	This study
MCZ151054	***Munidopsis hawaii* sp. nov.**	MZ543405	OP942425	OP947662	This study
MNHN-IU-2016-2446	*Munidopsis amapa*	OL685171	OL681876	ON858143	Rodríguez-Flores, Macpherson & Machordom (2022b)/this study
MNHN-IU-2014-23830	*Munidopsis balconi*	OL685172	OL681877	ON858142	Rodríguez-Flores, Macpherson & Machordom (2022b)/this study
MNHN-IU-2014-13822	*Munidopsis barbarae*	MG979479	MG979472	ON858166	Rodríguez-Flores, Macpherson & Machordom (2018)/this study
MNHN-IU-2014-13823	*Munidopsis barbarae*	MG979478	MG979471	ON858167	Rodríguez-Flores, Macpherson & Machordom (2018)/this study
MNHN-IU-2016-2560	*Munidopsis corniculata*	MG979480	MG979473	ON858169	Rodríguez-Flores, Macpherson & Machordom (2018)/this study
MNHN-IU-2013-19128	*Munidopsis corniculata*	MG979481	MG979474	ON858168	Rodríguez-Flores, Macpherson & Machordom (2018)/this study
MBM189200	*Munidopsis dispar*	MT901051	MT896787		Dong, Gan & Li, 2021
MBM286629	*Munidopsis dispar*	MT901052	MT896788		Dong, Gan & Li, 2021
MBM286630	*Munidopsis dispar*	MT901053	MT896789		Dong, Gan & Li, 2021
MCZ151064	*Munidopsis dispar*	OP933766	OP942426	OP947663	This study
MBM286648 1	*Munidopsis guochuani*	MT901056	MT896792		Dong, Gan & Li, 2021
MBM286648 2	*Munidopsis guochuani*	MT901057	MT896793		Dong, Gan & Li, 2021
MBM286648 3	*Munidopsis guochuani*	MT901058	MT896794		Dong, Gan & Li, 2021
MBM286647	*Munidopsis guochuani*	MT901055	MT896791		Dong, Gan & Li, 2021
MBM189201	*Munidopsis guochuani*	MT901054	MT896790		Dong, Gan & Li, 2021
MCZ151038	*Munidopsis guochuani*	MZ543406	OP942427	OP947664	This study
MBM189188	*Munidopsis kexueae*	MT901050	MT896786		Dong, Gan & Li, 2021
NIWA73782	*Munidopsis papanui*	OP933765			This study
MNHN-IU-2016-2365	*Munidopsis pholidota*	OL685170	OL681875	ON858141	Rodríguez-Flores, Macpherson & Machordom (2022b)/this study
MNHN-IU-2013-18964	*Munidopsis senticosa*	MG979483	MG979476	ON858173	Rodríguez-Flores, Macpherson & Machordom (2018)/this study
MNHN-IU-2013-18962	*Munidopsis senticosa*	MG979482	MG979475	ON858172	Rodríguez-Flores, Macpherson & Machordom (2018)/this study
MNHN-IU-2013-18901	*Munidopsis squamosa*	OL685169	OL681874	ON858140	Rodríguez-Flores, Macpherson & Machordom (2018)/this study

### New taxa

The electronic version of this article in Portable Document Format (PDF) will represent a published work according to the International Commission on Zoological Nomenclature (ICZN), and hence the new names contained in the electronic version are effectively published under that Code from the electronic edition alone. This published work and the nomenclatural acts it contains have been registered in ZooBank, the online registration system for the ICZN. The ZooBank LSIDs (Life Science Identifiers) can be resolved and the associated information viewed through any standard web browser by appending the LSID to the prefix http://zoobank.org/. The LSID for this publication is: [urn:lsid:zoobank.org:pub:1ADAB15A-E85C-481C-B5BA-C86D1B9A6868]. The online version of this work is archived and available from the following digital repositories: PeerJ, PubMed Central SCIE and CLOCKSS.

## Results

Three species of *Munidopsis* were collected from the vicinity of the Hawaiian Islands: *M. dispar* Dong, Gan & Li, 2021, *M. guochuani* Dong, Gan & Li, 2021 and a new species, (*M. hawaii* **sp. nov.**), which is described using integrative taxonomy. Furthermore, using the images obtained by ROVs we found a total of six species of Munidopsidae from seamounts in the vicinity of the Hawaiian Archipelago.

### Munidopsid images captured by ROVs

Video and/or still images of munidopsid species available online (NOAA Ocean Exploration Benthic Deepwater Animal Identification Guide, http://oceanexplorer.noaa.gov/okeanos/animal_guide/animal_guide.html) were examined, after selecting the region of interest (from Midway Atoll to Hawaiian Archipelago and including Johnston Atoll) and using the keyword ‘squat lobster’. Information regarding the expedition, transect site, date, and depth at which the video was filmed is included online. We only list taxa that could be identified to species or close to a species (identified as aff.). A summary of the observed munidopsids is provided in Table S1. *Munidopsis albatrossae*, *M. bairdii-arietina* complex, *Galacantha* aff. *rostrata* and *M. guochuani* were observed in one or several of the images captured with ROVs in the Johnston Atoll, PMNM, and near the Hawaiian Archipelago. These species present a unique morphotype compared to their relatives, which makes them relatively easy to distinguish (*e.g*., a longitudinal row of prominent spines and tubercules on the carapace in *G. rostrata*, rostrum broadly triangular and carapace comparatively wider in *M. albatrossae*). Several other species of *Munidopsis* can be spotted in these videos, however, they cannot be identified from images alone.

**Systematics**

**Galatheoidea Samouelle, 1819**

**Munidopsidae Ortmann, 1898**

***Munidopsis dispar*** Dong, Gan & Li, 2021

Figure 1A

**Figure 1:**
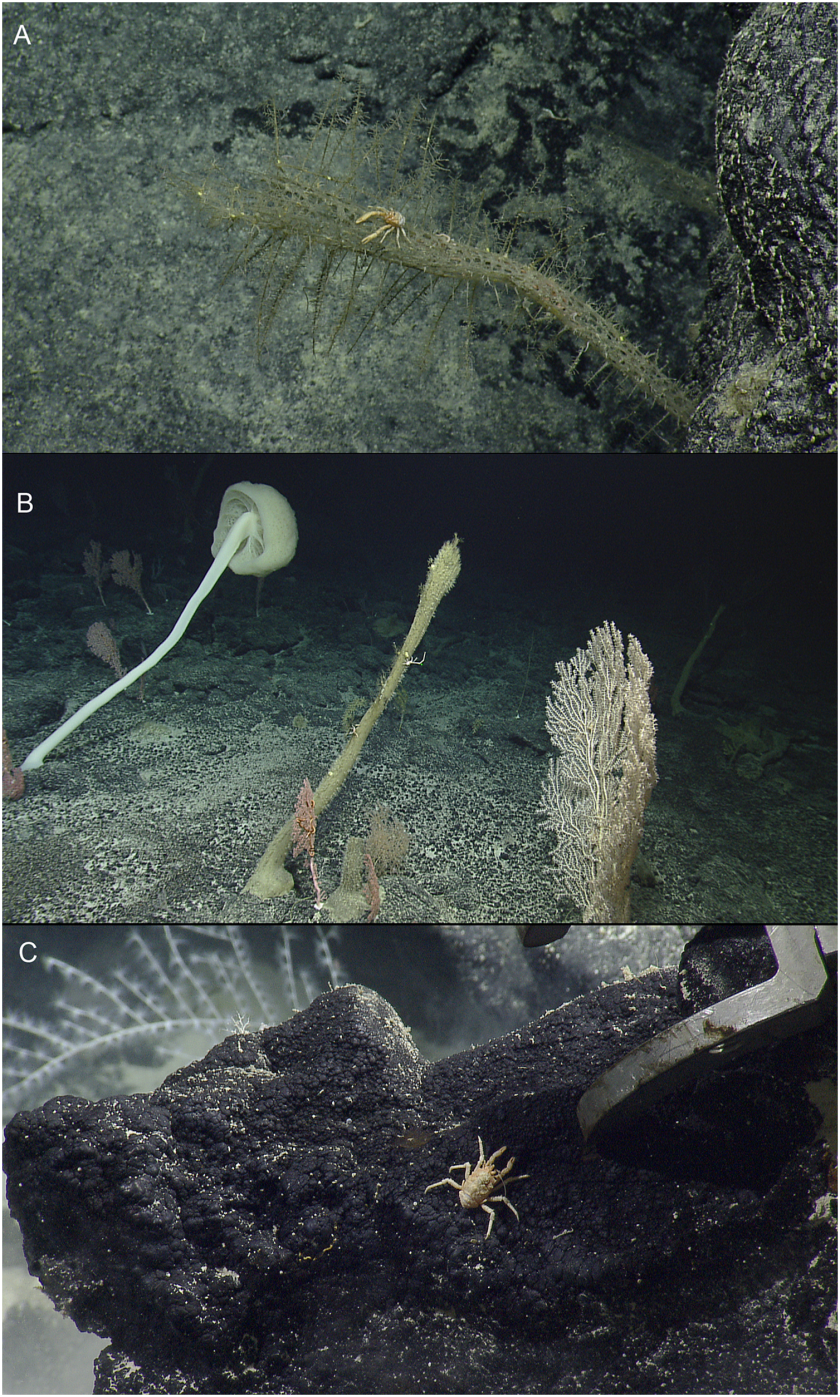
*In situ* images of deep-sea squat lobsters. (A) *Munidopsis dispar* Dong, Gan & Li, 2021, Off Hawaii’i, Stn NA101-060-01-B (MCZ IZ-151064). (B) *Munidopsis guochuani Dong, Gan & Li, 2021*, Off Hawai’i, Stn NA101-080 (MCZ IZ-151038-9). (C) *Munidopsis hawaii* sp. nov., Off Hawai’i, Stn NA101-070-01 (MCZ IZ-151054). Images provided by Natilus Live Ocean Exploration Trust.

**Material examined.** USA: Off Hawai’i, coll. EV Nautilus, cruise NA101-Hawaii, dive H1721, Stn NA101-060-01-B, 24 September 2018, 26.70855°, −166.98789°, 2,072 m: 1 M 6.6 mm (MCZ IZ-151064).

**Distribution.** Previously recorded from seamounts located in the Mariana Trench and the Caroline Plate at 1,246–1,458 m depth. Collected from off Hawaiian Archipelago at 2,072 m.

**Habitat.** The species was previously found associated with corals, (*Chrysogorgia* Duchassaing & Michelotti, 1864 and *Bathypathes* Brook, 1889) (Dong, Gan & Li, 2021). The specimen here examined was attached to a sponge of the genus *Walteria* Schulze, 1886.

**Genetic data.** COI, 16S rRNA, and 28S rRNA.

**Remarks.** This species is characterized by having tubercles covering the dorsal surface of the carapace, triangular epigastric processes, and a narrow triangular rostrum. Intraspecific genetic distances between these specimens and populations from the West Pacific ranged from 0.25% to 0.6% for the COI partial gene.

***Munidopsis guochuani*** Dong, Gan & Li, 2021

Figures. 1B and S1.

**Material examined.** USA: Off Hawai’i, coll. EV Nautilus, cruise NA101-Hawaii, dive H1723, Stn NA101-080, 26 September 2018, 25.55541°, –164.20344°, 1,945 m: 1 M 9.7 mm (MCZ IZ-151038**)**.— USA: Off Hawai’i, coll. EV Nautilus, cruise NA101-Hawaii, dive H1723, Stn NA101-080, 26 September 2018, 25.55543°, −164.20345°, 1,945 m: 1 F 11.1 mm (MCZ IZ-151039).

**Distribution.** Previously recorded from Magellan Seamounts and a seamount located on the Caroline Plate in the western Pacific, between 1,332 and 1,458 m depth. Collected from off Hawaiian Archipelago at 1,945 m.

**Habitat**. This species is usually found on hydrocorals but also on the bottom of the seafloor (Dong, Gan & Li, 2021). Both male and female here examined were found clinging to a hydroid.

**Genetic data.** COI, 16S rRNA, and 28S rRNA.

**Remarks.** The species is characterized by having an elongated and slender rostrum, P1 distinctly more than twice carapace length, coxae clearly visible dorsally, and carapace covered by small tubercles or spines and with rows of prominent spines on the dorsomidline and lateral margins. This species resembles morphologically *M. sarissa* Lin, Osawa & Chan, 2007 from Taiwan in having the coxae clearly visible dorsally and a very elongated P1.

Genetic distances between specimens and populations from the West Pacific ranged from 0.7% to 1.5% for the COI partial gene. Multiple images from ROVs of this species suggest that it is an abundant species in cold-water coral reefs.

***Munidopsis hawaii* sp. nov.**

Zoobank LSID: urn:lsid:zoobank.org:act:788F50D0-F395-4E48-A99E-F48821E4C737.

**Figure 2:**
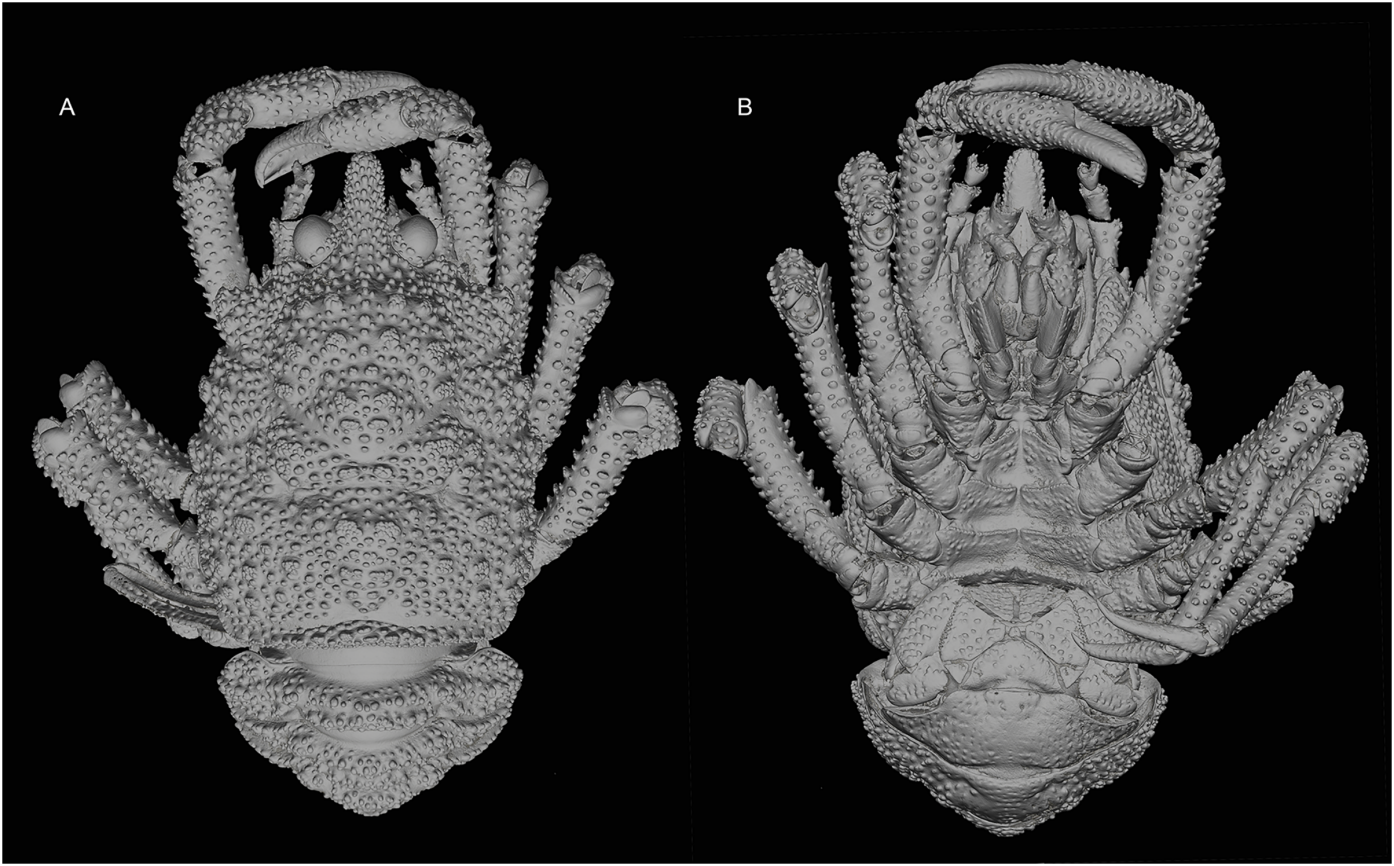
General habitus of the new species. 3D-renderings of micro-computed tomography x-ray images. *Munidopsis hawaii* **sp. nov.,** M 7.9 mm, holotype (MCZ IZ-151054) off Hawai’i. (A) General habitus dorsal view. (B) General habitus ventral view. 3D images are available from MCZbase.

**Figure 3:**
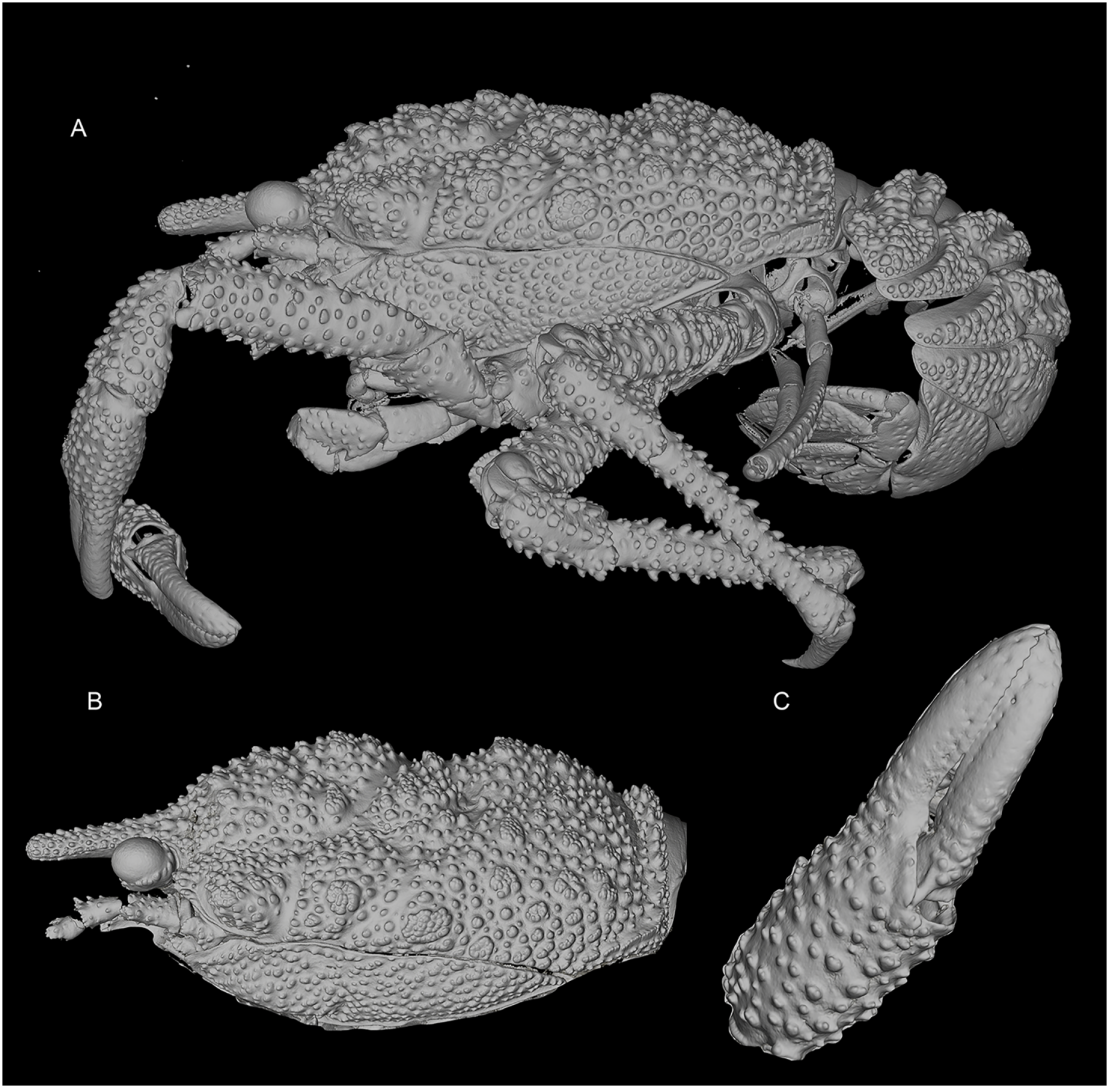
General habitus and details of the new species. 3D-renderings of micro-computed tomography x-ray images. *Munidopsis hawaii* **sp. nov.,** M 7.9 mm, holotype (MCZ IZ-151054) off Hawai’i. (A) General habitus lateral view. (B) Carapace, lateral view. (C) Detail of palm and fingers of left P1, outer view. 3D images are available from MCZbase.

**Figure 4:**
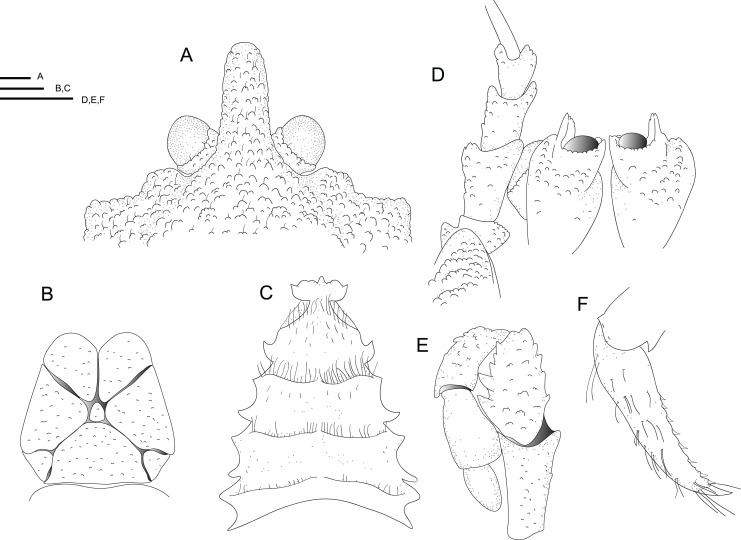
Line drawings of the new species. Line drawings of *Munidopsis hawaii* **sp. nov.,** M 7.9 mm, holotype (MCZ IZ-151054) off Hawai’i. (A) Anterior part of the carapace with ocular peduncles, dorsal view. B. Telson. (C) Sternal plastron. (D) Cephalic region, showing antennular and antennal peduncles and anterior portion of pterygostomian flap, ventral view. (E) Left Mxp3, lateral view. (F) Left P4 dactylus, lateral view. Scales = 1 mm.

Figs. 1C and 2–4, S2.

**Material examined.** *Holotype*. USA: Off Hawai’i, coll. EV Nautilus, cruise NA101-Hawaii, dive H1722, Stn NA101-070-01, 26 September 2018, 25.97258°, −164.74131°, 2,371 m: M 7.9 mm (MCZ IZ-151054).

**Etymology.** Named after the Hawaiian Archipelago since this species would constitute the first munidopsid species described from the area. The name is a substantive in apposition.

**Diagnosis.** Carapace and abdomen dorsally covered with denticulate tubercles and granules. Carapace with dorsal deep furrows, cervical grooves distinct. Rostrum narrow, with a blunt tip. Frontal margins slightly concave. Orbit slightly excavated, outer orbital angle with a blunt lobe. Anterolateral angle bluntly produced. Branchial margin unarmed. Abdominal somites unarmed. Telson divided into eight plates. Sternite 3 anterolaterally produced, anterior margin with a median acute lobe flanked by two rounded lobes; sternite 4 anteriorly narrowly subtriangular. Eyes unmovable, unarmed, epistomial spine absent. Article 1 of antennule with well-developed dorsolateral process, mesially concave. Article 1 of antenna with distomesial blunt process, distolateral spine short, broad. Mxp3 merus subrhomboidal in lateral view, extensor margin distally unarmed. P1 moderately slender, with granules and tubercles, unarmed; fixed finger without denticulate carina on distolateral margin. P2–4 moderately slender, with numerous granules and tubercles, unarmed; meri cylindrical in cross section; dactyli slender, curving; flexor margin with obsolescent teeth on distal two-thirds. Epipods present on P1–3.

**Description**. Carapace: Slightly longer than broad, widest at midlength; moderately convex from side to side. Dorsal surface covered with denticulate tubercles and granules, each tubercle and granule with few short setae; hepatic and anterior branchial areas with granules and some acute tubercles. Regions well delineated by furrows including distinct anterior and posterior cervical grooves. Gastric region slightly elevated. Posterior margin preceded by elevated ridge. Rostrum narrow, lateral margins subparallel, with a blunt tip, slightly directed downwards, covered with denticulate tubercles; 0.3 times as long as remaining carapace, 1.6 times as long as broad. Frontal margin slightly concave behind ocular peduncle, outer orbital angle slightly produced into blunt lobe above antennal peduncle. Lateral margins slightly convex; anterolateral angle bluntly produced; branchial margin slightly convex. Pterygostomian flap surface covered by granules and tubercles, anterior margin blunt, unarmed.

Sternum: Longer than broad, maximum width at sternite 7. Sternite 3 broad, 2.6 times wider than long, anterolaterally produced, anterior margin with a median acute lobe flanked by two rounded lobes. Sternite 4 narrowly elongate anteriorly; surface depressed in midline, with granules; greatest width 2.5 times that of sternite 3, and 1.6 times wider than long.

Abdomen: Unarmed; tergites 2–4 with two elevated transverse ridges, dorsal surfaces entirely tuberculate; tergites 5–6 with only anterior transverse ridge; tergite 6 with weakly produced posterolateral lobes and nearly transverse posteromedian margin. Telson composed of eight plates; 1.3 times as wide as long.

Eye: Eyestalk unmovable; peduncle short and fixed, covered by denticles, shorter than cornea length, wider than cornea width; cornea globular, unpigmented; epistomial spine absent.

Antennule: Article 1 of peduncle with well-developed dorsolateral process, mesially concave; ventral surface tuberculate.

Antenna: Peduncle overreaching distal margin of cornea by nearly full length of articles 3 and 4. Articles covered by denticulate tubercles. Article 1 with distomesial blunt process; distolateral spine broad. Articles 2–4 unarmed. Flagellum longer than carapace.

Mxp3: Ischium as long as merus measured on extensor margin, with distal flexor and extensor spines. Crista dentata finely denticulate. Merus with five spines along flexor margin, proximal two larger; row of four small spines and blunt distal process on extensor margin. Carpus, propodus and dactylus unarmed.

P1: Moderately slender, covered by tubercles and granules, 1.9 × longer than carapace. Merus 1.8 × carpus length, with some distal stout spines. Carpus 1.5 × longer than broad, with rows of spines on mesial and lateral margins and some distal stout spines. Palm unarmed, stout, slightly longer than carpus, 1.2 × longer than broad. Fingers unarmed, 1.1 × longer than palm, opposable margins straight, not gaping, spooned distally, fixed finger without denticulate carina on distolateral margin.

P2–4: Moderately slender, overreaching end of P1, covered by granules and tooth-like tubercles devoid of setae, cylindrical in cross section, decreasing in size posteriorly. P2 merus moderately slender, 0.5 × carapace length, nearly 3.5 × longer than high and 1.2 times length of P2 propodus. P2–4 meri decreasing in length posteriorly (P3 merus 0.9 length of P2 merus, P4 merus 0.8 length of P3 merus); extensor margin of P2–4 meri entirely furnished with tubercles, distal part flattish with tubercles; flexor margin denticulate, unarmed; carpi tuberculate, unarmed, granulate carina along lateral side. P2–4 propodi 5.0–6.0 × longer than broad, cylindrical in cross section, armed with numerous tubercles on flexor and extensor margins and dorsal and ventral surfaces. Dactyli 0.5–0.6 × length of propodi; distal claw short, moderately curved; flexor margin distally curved, with 6–7 obsolescent teeth only on distal two-thirds, decreasing in size proximally, ultimate tooth closer to penultimate tooth than to dactylar angle.

Epipods present on P1–3.

**Coloration:** Body light orange, whitish eyes.

**Distribution**. Seamount off Hawai’i, at around 2,370 m.

**Habitat**. Found in the conglomerated sediment.

**Genetic data.** COI, 16S rRNA, and 28S rRNA.

**Remarks.** *Munidopsis hawaii* **sp. nov**. belongs to the group of species having epipods on P1–3, lateral margins of the carapace unarmed, rostrum narrow, not acute at tip, frontal margin concave behind ocular peduncle, ocular peduncle short and fixed, unarmed, without eye-spine or lobes, antennal articles 2 and 3 unarmed, telson composed of eight plates, and spines on flexor margin of P2–4 dactyli minute or obsolescent. This group of species includes *Munidopsis cidaris* Baba, 1994 from the West Pacific, and other species from the West Atlantic: *M. espinis* Benedict, 1902. The new species can be easily distinguished from the others by the following characters:

– *Munidopsis hawaii* **sp. nov.** is densely covered with tubercles and denticles on carapace, abdomen, and pereopods, whereas the other species have a smooth or at most granulated carapace and abdomen.
– *Munidopsis hawaii* **sp. nov.** has a rostrum with subparallel lateral margins, whereas the other species have a subtriangular rostrum with distinctly convergent lateral margins.
– The anterolateral angles of the carapace are less acute in *Munidopsis hawaii* **sp. nov.** compared to the other species from the group.

*Munidopsis hawaii* **sp. nov**. also closely resembles other West Pacific species with numerous tubercles or denticles on the carapace, abdomen and pereopods and with unarmed lateral carapace margins: *M. laevisquama* Lin & Chan, 2011 from Taiwan, *M. papanui* Schnabel & Bruce, 2006 from New Zealand, *M. sonne* Baba, 1995 from North Fiji, and *M. tuberosa* Osawa, Lin & Chan, 2008 from Taiwan and South China Sea. However, the new species can be easily distinguished from these species using the following distinctive characters:

– The eye has prominent distomesial protuberances in *M. papanui*, but those protuberances are absent in *M. hawaii* sp. nov. and other species.
– *Munidopsis hawaii* **sp. nov.**, (as *M. laevisquama, M. papanui, M. sonne* and *M. tuberosa*) has epipods on P1–3 whereas *M. dispar* lacks epipods on all pereopods. The tubercles on the carapace are more acute and triangular in *M. dispar* than in the new species. The rostrum is dorsally carinated in *M. dispar*, whereas there is no dorsal carina in the new species.
– The new species have the rostrum margins parallel whereas these margins are convergent in *M. dispar* and *M. papanui* or constricted between eyes in *M. laevisquama, M. sonne* and *M. tuberosa*. The new species has a blunt rostral tip (not acute) whereas the rest of the species have a subtriangular rostrum with a rather acute tip.
– The new species only has obsolescent teeth on the distal two-thirds of flexor margins of P2–4 dactyli, whereas these teeth are distinct in *M. dispar*, *M. laevisquama*, *M. papanui*, and *M. tuberosa*.

Sequence divergence between *Munidopsis hawaii* sp. nov., *M. papanui*, and *M. dispar* is up to 13% for COI and 6% for 16S. Unfortunately, we do not have molecular data for the other species.

### Phylogenetic analyses and genetic distances

**Figure 5:**
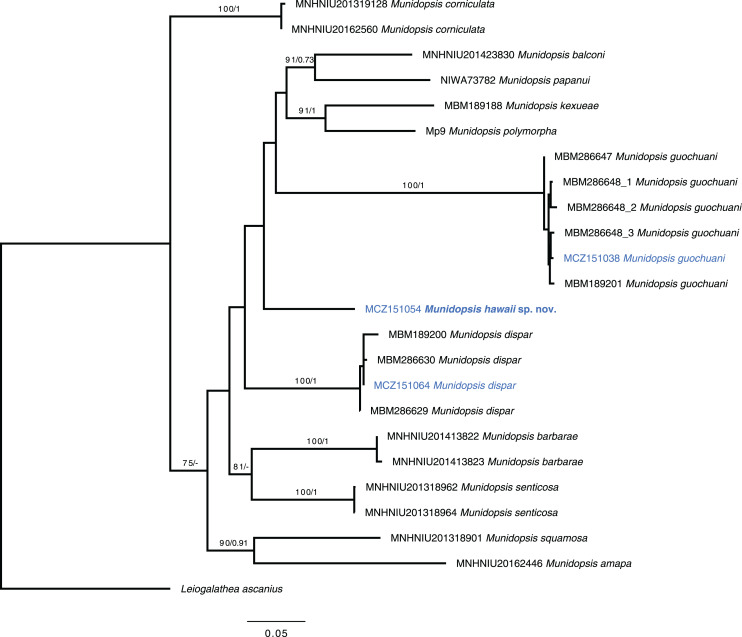
Phylogenetic tree of the new species and relatives. Phylogenetic hypothesis of the concatenated data set (COI + 16S rRNA + 28S rRNA) resulting from the ML analysis. Tip label in bold indicates new taxa. Asterisks indicate supported nodes: ML bootstrap support >70%/Bayesian posterior probability >0.95.

*Munidopsis guochuani* and *M. dispar* could be unequivocally aligned with reference sequences (Dong, Gan & Li, 2021), presenting intraspecific distances under 1.5% for the COI barcode in both cases. The new species constitute a lineage that diverges on more than 13.7% for this marker with other morphologically similar species analyzed within *Munidopsis* and does not appear to align with any clades established for other congeners (Rodríguez-Flores, 2021). The divergence among the new species and the other analyzed species ranged from 13.7% to 19.0% for the COI partial gene, 6.4% to 13% for the 16S, and 0.3% to 3% for the fragment of the nuclear gene 28S. The systematic position of the new species is unsolved with the data available (Fig. 5).

## Discussion

*In situ* photos and videos of live organisms resulting from deep-sea exploration provide an invaluable source of data for the study of the distribution, ecology, and behavior of species that are poorly known. Thanks to images collected with ROVs, we can observe how species behave, and where they live and learn about associations between invertebrates in the deep sea (Wicksten, 2020). However, it is also essential to collect specimens for several reasons. For instance, most squat lobster species are difficult to identify without a closer look at specific morphological characters such as mouth parts or other appendages, often requiring the use of a microscope. Also, there are estimates suggesting that thousands of species of squat lobsters are waiting to be discovered and described (Appeltans et al., 2012). This fact, together with estimates that around 95% of the deep sea is still unexplored (Schnabel et al., 2020), indicates that most of the species of squat lobsters seen in ROVs images, at least in under-sampled regions, may correspond to undescribed taxa. Examination of some diagnostic characters, such as the presence of epipods is impossible by just examining images taken from ROVs (Macpherson, 2007; Wicksten, 2020). Moreover, morphological convergence is a common trend in squat lobsters, so most characters useful for distinguishing species, including the shape of a rostrum, carapace tuberculation, and ornamentation, are shown to be frequently homoplastic (Machordom et al., 2022; Rodríguez-Flores, Macpherson & Machordom, 2022b).

The new species here described is morphologically similar to other species of *Munidopsis* from the West Pacific in terms of the ornamentation of the carapace: *e.g*., *M. dispar, M. laevisquama, M. papanui*, *M. sonne*, and *M. tuberosa* among others. However, some of them represent highly divergent and remote lineages according to molecular characters (Dong, Gan & Li, 2021; Rodríguez-Flores, Macpherson & Machordom, 2022b). The genus *Munidopsis* is one of the most diverse groups within squat lobsters (Baba et al., 2008) and one of the most abundant decapods at abyssal depths (Macpherson et al., 2010). *Munidopsis* is characterized by considerable morphological diversification, with an extreme variety of morphotypes, sizes, and ornamentation among other characteristics (Rodríguez-Flores, Macpherson & Machordom, 2022b). For that reason, multiple genera and/or subgenera were previously considered; however, overlapping morphologies and unresolved morphological delimitation resulted in the retention of only one single genus *Munidopsis* (Chace, 1942; Baba, 2005; Macpherson, 2007). Molecular phylogenies indicate that the subfamily classification within Munidopsidae is not supported, *Munidopsis* is para- or polyphyletic, and some of the recovered clades correspond to formerly recognized genera or subgenera (Ahyong, Andreakis & Taylor, 2011; Rodríguez-Flores, 2021) a case that has been also seen in other highly diverse squat lobsters (Machordom et al., 2022). However, more taxa should be added to fully solve how many genera include the deep-sea squat lobsters’ tree of life. Basic taxonomic research is essential for this purpose, like discovering new species that could correspond to highly divergent lineages within Munidopsidae, as the species here described. Maximizing taxon sampling, to include DNA sequence data as well as illustrating and/or redescribing insufficiently described old species (*e.g*., Dong et al., 2019; Dong, Gan & Li, 2021; Rodríguez-Flores, Macpherson & Machordom, 2022b), should be a priority to resolve the real diversity of the group. In this sense, MicroCT imaging allows getting high-resolution images of the external and internal anatomy without compromising the integrity of the specimen. Several authors are using this technique for taxonomic research in crustaceans since it can be also used for old material, and it did not require staining for external morphology (*e.g*., Koch et al., 2017, Landschoff et al., 2018; Ng et al., 2021). 3D models of the specimen are available in databases, so in the long term, they can be a source for morphological comparison among species. Also, it is an extremely useful tool to study the patterns carapace ornamentation.

Prior to this study, the deep-sea squat lobster family Munidopsidae was not formally recorded from Hawaiian waters and surroundings. However, the NOAA database included multiple dive summary reports with sightings of squat lobsters in the genus *Munidopsis* from the area (*e.g*., Kennedy & Mcletchie, 2016). Most species of Brachyura and Anomura recorded from Hawai’i have shown to be species from the Indian or/and Pacific oceans, having a wide distribution (Castro, 2011). In contrast, most squat lobsters, Chirostyloidea, and several species of Galatheoidea, from Hawai’i have been only recorded in this area (Baba et al., 2008). The recent revisions of the genus *Phylladiorhynchus* Baba, 1969 (Schnabel & Ahyong, 2019; Rodríguez-Flores, Macpherson & Machordom, 2021) showed that *P. integrirostris* was indeed a species complex including regional species, and *P. integrirostris *sensu* stricto* is endemic to Hawai’i. Two recently described species of *Babamunida*, *Babamunida debrae* Baba, 2011 and *Babamunida kanaloa* Schnabel, Martin & Moffitt, 2009 have been only found in Hawaiian waters; and *Paramunida hawaiiensis* has been reported only from Hawai’i so far (Baba, 1981). In contrast to these shallow or shelf species, munidopsid species found with ROVs are widely distributed species or even with a cosmopolitan distribution. That is the case for *Munidopsis albatrossae*, *Galacantha rostrata*, *M*. *bairdii*-*arietina* complex, all of them bathyal/abyssal species with wide or cosmopolitan distribution (Baba et al., 2008). These species are usually observed wandering on the conglomerated sediment, although *Munidopsis bairdii-arietina* complex has been often captured hanging on corals or sponges. The recently described *Munidopsis guochuani* can be observed in a considerable number of ROVs videos and images, not only from off Hawai’i, but also from other seamounts across the West Pacific (*e.g*., https://www.ncei.noaa.gov/waf/okeanos-animal-guide/Galatheoidea068.html). *Munidopsis guochuani* can be observed associated with different species of hydrocorals but also on the seafloor (Dong, Gan & Li, 2021), and according to the frequency of the observations, it is most likely a frequent and abundant species. However, the fact that this distinctive species has been just recently described indicates that most of the deep-sea taxa remain to be discovered.

## Supplemental Information

10.7717/peerj.14956/supp-1 Supplemental Information 1 Live image of *Munidopsis guochuani*. *Munidopsis guochuani* Dong, Gan & X Li, 2021. Credits: Nautilus Live Ocean Exploration Trust.

10.7717/peerj.14956/supp-2 Supplemental Information 2 Live image of *Munidopsis hawaii*. *Munidopsis hawaii* sp. nov. Holotype (MCZ 151054). Credits: Nautilus Live Ocean Exploration Trust.

10.7717/peerj.14956/supp-3 Supplemental Information 3 Data of available ROV images of Munidopsidae in Hawaii and surroundings.

10.7717/peerj.14956/supp-4 Supplemental Information 4 Sequences 16S.

10.7717/peerj.14956/supp-5 Supplemental Information 5 Sequences 28S.

10.7717/peerj.14956/supp-6 Supplemental Information 6 Sequences COI.

## Additional Information and Declarations

### DNA Deposition

The following information was supplied regarding the deposition of DNA sequences: The group II intron/IEP sequences are available at GenBank: MZ543406 to MZ543405 and OL685169 to OL681874.

### Data Availability

The following information was supplied regarding data availability: The microCt scanning data is available at MorphoSource: Media 000478323, DOI 10.17602/M2/M478323.

### New Species Registration

The following information was supplied regarding the registration of a newly described species: Publication LSID: urn:lsid:zoobank.org:pub:1ADAB15A-E85C-481C-B5BA-C86D1B9A6868. Munidopsis hawaii sp. nov. LSID: urn:lsid:zoobank.org:act:788F50D0-F395-4E48-A99E-F48821E4C737.
