# Supplementary figures and images for: New records and species of deep-sea squat lobsters (Galatheoidea, Munidopsidae) from the Hawaiian Archipelago: an integrative approach using micro-CT and barcodes
Source: PeerJ. 2023 Mar 8;11:e14956. doi: 10.7717/peerj.14956 (PMC10007970; doi:10.7717/peerj.14956)

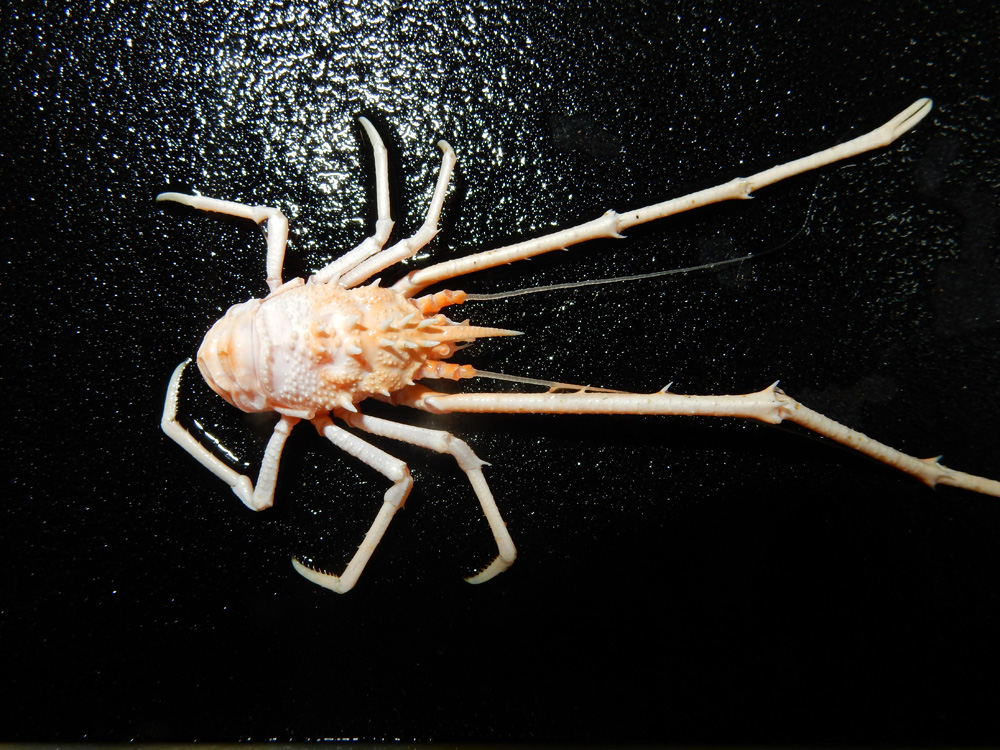

Supplement: Supplemental Information 1 — Munidopsis guochuani Dong, Gan & X Li, 2021. Credits: Nautilus Live Ocean Exploration Trust. [file peerj-11-14956-s001.jpg]

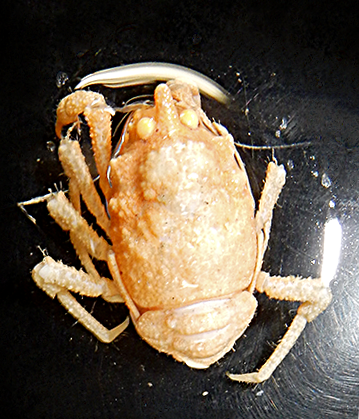

Supplement: Supplemental Information 2 — Munidopsis hawaii sp. nov. Holotype (MCZ 151054). Credits: Nautilus Live Ocean Exploration Trust. [file peerj-11-14956-s002.tif]
